# Development of Optogenetic Dual-Switch System for Rewiring Metabolic Flux for Polyhydroxybutyrate Production

**DOI:** 10.3390/molecules27030617

**Published:** 2022-01-18

**Authors:** Sumeng Wang, Yue Luo, Wei Jiang, Xiaomeng Li, Qingsheng Qi, Quanfeng Liang

**Affiliations:** 1 State Key Laboratory of Microbial Technology, National Glycoengineering Research Center, Shandong University, Jinan 250100, China; smwang001@126.com (S.W.); m18702537725@163.com (Y.L.); jw841813831@163.com (W.J.); Lleky0713@163.com (X.L.); 2 CAS Key Lab of Biobased Materials, Qingdao Institute of Bioenergy and Bioprocess Technology, Chinese Academy of Sciences, Qingdao 266101, China

**Keywords:** optogenetic, dynamic regulation, metabolic engineering, cell growth, polyhydroxybutyrate (PHB) production

## Abstract

Several strategies, including inducer addition and biosensor use, have been developed for dynamical regulation. However, the toxicity, cost, and inflexibility of existing strategies have created a demand for superior technology. In this study, we designed an optogenetic dual-switch system and applied it to increase polyhydroxybutyrate (PHB) production. First, an optimized chromatic acclimation sensor/regulator (RBS10–CcaS#10–CcaR) system (comprising an optimized ribosomal binding site (RBS), light sensory protein CcaS, and response regulator CcaR) was selected for a wide sensing range of approximately 10-fold between green-light activation and red-light repression. The RBS10–CcaS#10–CcaR system was combined with a blue light-activated YF1–FixJ–PhlF system (containing histidine kinase YF1, response regulator FixJ, and repressor PhlF) engineered with reduced crosstalk. Finally, the optogenetic dual-switch system was used to rewire the metabolic flux for PHB production by regulating the sequences and intervals of the citrate synthase gene (*gltA*) and PHB synthesis gene (*phbCAB*) expression. Consequently, the strain RBS34, which has high *gltA* expression and a time lag of 3 h, achieved the highest PHB content of 16.6 wt%, which was approximately 3-fold that of F34 (expressed at 0 h). The results indicate that the optogenetic dual-switch system was verified as a practical and convenient tool for increasing PHB production.

## 1. Introduction

Several natural cells have light-sensing proteins that respond to red, green, blue, ultraviolet, or near-infrared (NIR) [1,2,3]. Photosensory systems can be categorized into one-component (e.g., EL222 [4], YtvA [5], and VVD [6]) or two-component (e.g., CcaS–CcaR [7,8,9], YF1–FixJ [10], and Cph8–OmpR [11]) systems. A two-component system comprises a sensory histidine kinase (HK) and a response regulator (RR). The activities of the HK and RR are directed by the phosphorylation or dephosphorylation that occurs in response to different wavelengths of illumination [7,12]. CcaS belongs to the cyanobacteriochrome family of proteins, which respond to green and red light through the combination of the N-terminal GAF domain (cyclic GMP phosphodiesterase, adenylyl cyclase, FhlA) with a phycocyanobilin chromophore. CcaR is a member of the OmpR class, and it contains C- and N-terminal domains that function as a DNA binder and receiver, respectively [7]. The blue light-sensing YF1–FixJ system was constructed by replacing the PAS (period/aryl hydrocarbon receptor nuclear translocator/single-minded) sensory domain of *Bradyrhizobium japonicum* FixL–FixJ with the LOV domain of the *Bacillus subtilis* photosensor YtvA [10]. YF1 is repressed in blue light and activated in darkness through the combination of the LOV domain with flavin mononucleotide (FMN). LexRO is a recombined light sensory protein in which the blue light sensory domain RsLOV from *Rhodobacter sphaeroides* is fused with the DNA-binding domain of the mutated *Escherichia coli* repressor LexA_408_ [13]. In a one-component system, a sensory domain can be activated or repressed directly by light or darkness inducing a conformational change [12]. For example, EL222 consists of the LOV sensory domain, Jα helix connector, and helix-turn-helix (HTH) domain effector. In the dark, the interaction of the FMN-connected EL222 LOV domain with HTH inhibits the binding of this domain with DNA. The LOV and HTH domains are released under blue light illumination and generate homodimerization to bind DNA for activation [4]. Optogenetics have frequently been applied to metabolic regulation in several microorganisms [11,14,15,16,17,18]. For example, Miyake et al. constructed a green-light inducible lytic system in *Synechocystis* sp. PCC 6803 through the CcaS/CcaR light sensory system [18]. Tandar et al. designed a light-inducible valve (CcaSR-*pgi* ver. 3) that can regulate the metabolic flux of the oxidative pentose phosphate (oxPP) and Embden–Meyerhof–Parnas (EMP) pathways by controlling the expression of *pgi*, which then controls EMP and oxPP flux distribution with alternating red and green light [19]. Two blue light-activated optogenetic systems, OptoEXP and OptoINVRT, were developed to produce isobutanol and 2-methyl-1-butanol in *Saccharomyces cerevisiae* [20]. Cell division can also be controlled through a combination of blue light and near-infrared (NIR) light activation systems, and it can then be used in acetoin and poly(lactate-co-3-hydroxybutyrate) production [21].

Polyhydroxybutyrate (PHB), the most common member of the polyhydroxyalkanoates (PHAs), has been synthesized using multiple microorganisms and has been identified as a biodegradable polymer [22,23,24]. In a PHB metabolic pathway, acetyl-CoA is a key precursor in PHB production [25]. Acetyl-CoA is also a vital metabolite that contributes to the tricarboxylic acid (TCA) cycle for maintaining cell growth [26]. The random distribution of acetyl-CoA in TCA and PHB flux greatly affects cell growth and PHB production. Unbounded acetyl-CoA flux in the TCA cycle can enhance cell growth but results in poor production. By contrast, although PHB production can be improved by enhancing the metabolic flux from the acetyl-CoA-to-PHB pathway, premature repression of the TCA cycle results in poor cell growth. The imbalanced allocation of acetyl-CoA to the TCA cycle and PHB pathway leads to limited PHB production. Dynamic regulation of the acetyl-CoA metabolic flux can be implemented to overcome competitive cell growth and PHB production. Previously, we developed several dynamic methods for regulating metabolic flux and enhancing PHB production in *E. coli*; they include an autoinduced AND gate, a native interspaced short palindromic repeats interference (CRISPRi) system, and quorum sensing-based (QS-based) bifunctional dynamic switches (QS switches) [26,27,28]. However, the activation and repression of gene expression in such strategies has proven inconvenient, especially when different sequences and intervals of gene expression must be regulated [20,29]. 

**Figure 1:**
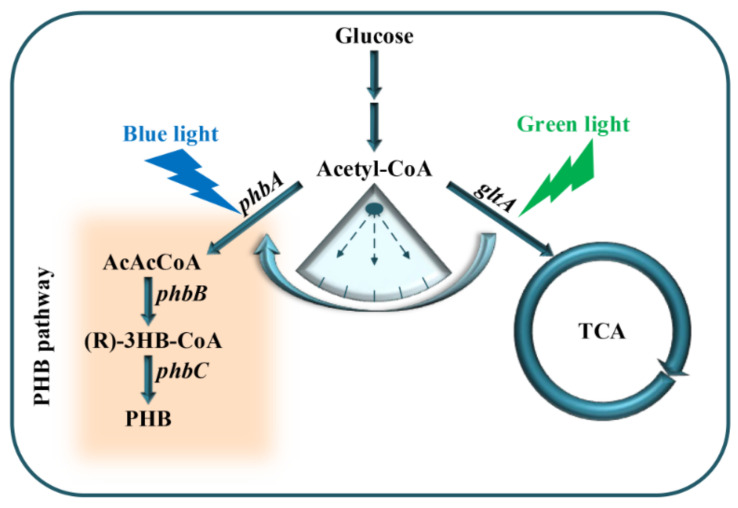
Dynamic regulation of PHB production using an optogenetic dual-switch system; *phbCAB*, PHB production gene cluster; *gltA*, citrate synthase used to catalyze acetyl-CoA and oxaloacetate into citrate; green light is used to activate *gltA* expression using RBS10–CcaS#10–CcaR; *phbCAB* is activated under blue light using YF1–FixJ–PhlF.

In the present study, cell growth and PHB production were optimized by dynamically regulating acetyl-CoA metabolic flux through the combination of the engineered RBS10–CcaS#10–CcaR and YF1–FixJ–PhlF with multiple expression sequences and time lags between *gltA* and *phbCAB* (Figure 1). Consequently, PHB content improved by approximately 11.5 wt% with the interval between *gltA* and *phbCAB* expression being controlled at 3 h. 

## 2. Materials and Methods 

### 2.1. Strains, Plasmids, Primers, and Culture Media

All strains, plasmids, and primers used in the present study are detailed in Tables S1–S3. *Escherichia coli* DH5α was used to construct plasmids, and *E. coli* TOP10 was used for characterization and PHB production. A Luria–Bertani (LB) medium containing 5 g/L yeast extract, 10 g/L tryptone, and 10 g/L NaCl was used for plasmid construction and characterization. A modified M9 medium consisting of 15.3 g/L Na_2_HPO_4_·12H_2_O, 3 g/L KH_2_PO_4_, 1 g/L NH_4_Cl, 1 g/L NaCl, 0.24 g/L MgSO_4_, 0.015 g/L CaCl_2_, 2 g/L yeast extract, and 20 g/L glucose was used for shake-flask PHB fermentation. 

### 2.2. Plasmid and Strain Construction

To construct pGX, CcaS was first amplified from pSR43.6 HN (pSR43.6), as described by Schmidl et al. [30]. The CcaS was then truncated through overlapping the N-terminal transmembrane helix with a GAF fragment (from base 1 to 663) with a fragmentary second linker region (L2) and a C-terminal HK domain (from base 1537 to 2262) to generate CcaS#10. Gene amplification and overlap were performed using Phanta HS Super-Fidelity DNA Polymerase, which was purchased from Vazyme Biotech (Nanjing, China). CcaR, *cpcG2* promoter, *sfgfp*, *ho1*, and *pcyA* were amplified from pHZ3.1 (pSR58.6) or pSR43.6 HN (pSR43.6), as described by Schmidl et al. [30]. The backbone of pGX was amplified from pSR43.6 HN (pSR43.6). CcaS#10, CcaR, *cpcG2* promoter, *sfgfp*, *ho1*, and *pcyA* were assembled into a pSR43.6 HN (pSR43.6) backbone to create pGX through the Gibson assembly method [31]. To construct a blue light-activated plasmid pYF1, *phlF*, YF1, and FixJ were amplified from the plasmids JFR1 and JFR2, as described by Fernandez-Rodriguez et al. [32]. The backbone of pYF1 was amplified from JFR1 and assembled with *phlF*, YF1, FixJ, and the reporter gene *rfp*. EL222, a transcription factor, from the marine bacterium *Erythrobacter litoralis* HTCC2594, was synthesized and codon-optimized in *E. coli* [4]. The fused EL222–*rfp* was assembled into a backbone and amplified from JFR1 to generate the plasmid pEL222 using 2X MultiF Seamless Assembly Mix (ABclonal Technology, Ltd. Wuhan, China). The light-triggered system LexRO was synthesized and codon-optimized in *E. coli*. Similarly, the fused LexRO–*rfp* was assembled with the backbone from JFR1 to form pLexRO. pDe-GX was constructed by removing the reporter gene *sfgfp*. To construct a PHB production plasmid, pYF1–PHB was created through the substitution of the *rfp* of the pYF1 with *phbCAB*. A ribosomal binding site (RBS) variant library (MAGGAGGWRDDTT) of CcaS#10 was created through the design of a pair of random primers with four degenerate bases (MWRD) to amplify different RBSs used in pGX to achieve different green-light and red-light fold changes. 

The strains #10-1–#10-10 were constructed by transforming pGX with different variant RBSs into *E. coli* TOP10 and were used to characterize CcaS#10–CcaR with variant RBSs. pYF1 was transformed into *E. coli* TOP10 to generate YF1–FixJ–PhlF to create a blue light-activated system. Similarly, *E. coli* TOP10 was transformed using pEL222 or pLexRO to construct an EL222 or LexRO strain. pGX and pYF1 were co-transformed into *E. coli* TOP10 to form GY for crosstalk analysis. The strains RBS30/31/32/33/34 were constructed by replacing the native promoter of *gltA* in the genome of *E. coli* TOP10 with P*cyG2*-B0030/31/32/33/34, respectively, along with the two transformed plasmids pDe–GX1 and pYF1–PHB. Replacement of the *gltA* promoter was performed through homologous recombination [33]. Briefly, λ-Red recombinase was expressed in the plasmid pTKRED and was used for homologous recombination through two long, designed homologous arms. The plasmid pCP20 was then used to remove the antibiotic-resistance gene. 

### 2.3. Characterization of Light-Sensing Systems

To characterize the light-sensing systems, the fluorescence intensity of the reporter genes *sfgfp* and *rfp* was monitored under various wavelengths of light using the Multi-Detection Microplate Reader (Synergy HT, BioTek, Winooski, VT, USA). Single colonies were cultivated in a 24-well microassay plate with 1 mL of LB medium supplemented with chloramphenicol or ampicillin at 37 °C for approximately 12 h. The precultures were then transferred to a 96-well microassay plate containing 200 μL of LB medium inoculated with 2% (*v*/*v*) for incubation at 37 °C. The culture was exposed to green light (at 1.96 W/m^2^), red light (at 3.92 W/m^2^), blue light (at 8.66 W/m^2^), or dark conditions (Figure S1a). The fluorescence intensity of the *rfp* was measured with excitation at 590 nm and emission at 645 nm. The fluorescence intensity of *sfgfp* was measured with excitation at 485 nm and emission at 528 nm. The fluorescence intensity was characterized relative to the optical density of *rfp* and *sfgfp* measured at 600 nm (OD_600_). To eliminate the effect of cells’ autofluorescence, the strain that did not contain reporters was concurrently cultivated, and autofluorescence was inhibited.

### 2.4. PHB Fermentation

Single colonies were cultivated in 10 mL LB medium containing 34 μg/mL chloramphenicol and 100 μg/mL ampicillin sodium at 37 °C overnight. The preculture was then transferred to a 300-mL shake flask containing 50 mL of M9 medium with 1% (*v*/*v*); the flask was supplemented with chloramphenicol and ampicillin and shaken at 220 rpm and at 37 °C for 54 h. When the glucose level was lower than 10 g/L, the flask was supplemented up to the approximate initial concentration of 20 g/L. During the fermentation procedure, ammonium hydroxide was used to maintain a pH of 7. In the present study, light-emitting diode belts with varying levels of illumination were attached to a thermostatic shaker. (Figure S1b). To activate the TCA pathway and repress the PHB pathway, green light was switched on, and blue light was switched off. Conversely, to repress the TCA pathway and activate the PHB pathway, blue light was turned on, and green light was turned off.

### 2.5. Analytical Method

OD_600_ was measured with a spectrophotometer (Shimadzu, Japan). To analyze PHB content, the fermentation culture was centrifuged at 10,625 g for 5 min. The resultant cell pellet was collected and washed twice with distilled water. The collected cells were lyophilized for 6 h and then pretreated with 150 μL of H_2_SO_4_ (98%, *w*/*w*), 850 μL of CH_3_OH, and 1 mL of CHCl_3_ at 100 °C for 1 h. Subsequently, 1 mL of deionized water was added and mixed completely. After the completion of phage separation, the CHCl_3_ layer was transferred to a new centrifuge tube for gas chromatography (GC) analysis of the PHB content. GC detection was conducted in accordance with the protocol used in our previous research [27]. A Shimadzu GC2010 gas chromatograph (Kyoto, Japan) equipped with an AOC-20i autoinjector and a Restek Rtx-5 column was used for PHB detection. The column temperature was initially set at 60 °C for 1 min, and it was increased to 230 °C at rate of 10 °C/min and maintained at 230 °C for 10 min. 

### 2.6. Statistical Analysis

The results are presented as means ± standard errors of the mean (SEMs). The differences between the means were evaluated using one-way analysis of variance or Tukey’s range test. A *p* value of <0.05 was regarded as statistically significant.

## 3. Results and Discussion 

### 3.1. Characterization and Screening of Optogenetic Elements

**Figure 2:**
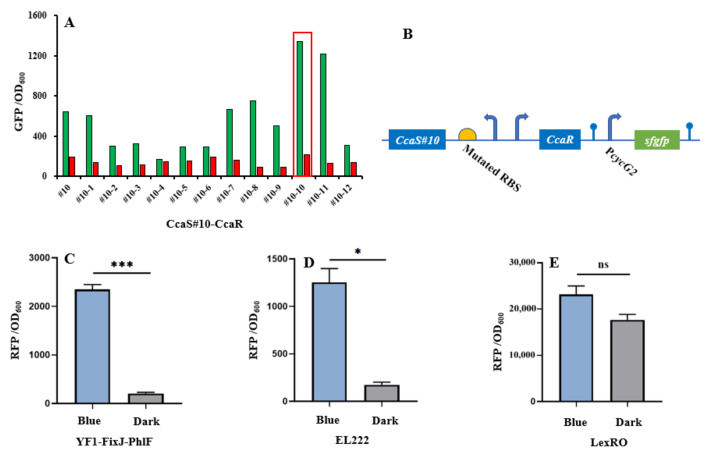
Characterization of light-trigged systems. (**A**) CcaS#10–CcaR system activated and repressed under green and red light, respectively. Strain #10 carrying CcaS#10–CcaR without RBS variants. Strains #10-1 to #10-12 containing variant RBS–CcaS#10–CcaR. (**B**) Light-sensing systems expressed in plasmids. (**C**–**E**) Characterization of three other blue light/darkness-sensing systems, YF1–FixJ–PhlF, EL222, and LexRO, under blue light or dark conditions. CcaS–CcaR and YF1–FixJ–PhlF were two-component systems; EL222 was classified as a one-component system. LexRO is a recombinant system that fuses the blue light sensory domain RsLOV with LexA408. Excluding the experiment (**A**) with single (*n* = 1) replication, other results (**C**–**E**) were calculated with three (*n* = 3) independent replications. Error bars represent means ± standard errors of the mean (SEMs). * *p* < 0.05; *** *p* < 0.001; ns, nonsignificance.

The CcaS–CcaR system consists of the light sensory protein CcaS and the response regulator CcaR and responds to 535-nm green and 672-nm red light [11]. Under green light illumination, CcaS is activated through autophosphorylation. The phosphate is then rapidly transferred to the CcaR for activating gene transcription with the promoter P*_cycG2_* [7,19]. Previously, a PAS domain-shortened CcaS#10 was designed to increase response ranges and reduce expression leakage under red light [8,9]. In this study, we used CcaS#10–CcaR as the initial light sensory system to allow for further study. To broaden the dynamic range of CcaS#10–CcaR, a small RBS variant library (MAGGAGGWRDDTT) was created through the replacement of four degenerate bases (Figure 2b). The heterologous genes heme oxygenase1(*ho1*) and ferredoxin (Fd) oxidoreductase (*pcyA*) were introduced to the plasmid pGX to synthesize phycocyanobilin with the aim of promoting a CcaS response [19]. The variant RBS was used to translate CcaS#10–CasR, and the translation of reporter gene *sfgfp* was controlled using the promoter P*cysG2* in the plasmid pGX. The characterization of variant RBSs was performed by observing the fluorescence intensity of *sfgfp* relative to OD_600_ under green- or red-light illumination in *E. coli* TOP10. Through subtraction of the autofluorescence of cells, strain #10-10, which harbored RBS10–CcaS#10–CcaR (optimized RBS sequence: aaggaggaaagtt), exhibited the greatest increase in dynamic range—an approximately 10-fold increase; this increase was higher than that achieved by strain #10, which contained CcaS#10–CcaR and for which the increase was approximately 3-fold (Figure 2a). Therefore, the optimized RBS10–CcaS#10–CcaR was utilized in follow-up experiments. 

To introduce another light-regulated system, we studied three photosensory systems in *E. coli*: YF1–FixJ, EL222, and LexRO [10,13,34]. Of these systems, EL222 and LexRO could directly activate and repress gene expression under blue light and dark conditions [10,13,34]. In darkness, blue light sensory domain RsLOV dimerizes and binds the operator to repress promoter activity. Under blue light conditions, dimerized RsLOV dissociates into monomers and leads to promoter activation [13]. For EL222, in the dark, the interaction of LOV and HTH domains results in an inability to bind DNA. Upon blue light exposure, HTH is released and homodimerized to bind the DNA sequence for promoter activation [4].By contrast, native YF1–FixJ is activated under dark conditions but repressed under blue light [10]. To construct a blue light-activated YF1–FixJ system, the repressor protein PhlF was introduced into the YF1–FixJ system to create YF1–FixJ–PhlF [32]. The regulation mechanism of YF1–FixJ–PhlF is explained as follows: Translation of *phlF* is controlled by a light-sensing promoter; the regulated gene is placed under the PhlF-binding operator. Under dark conditions, the expression of *phlF* is activated by the histidine kinase YF1-response regulator FixJ system, and then bound to the *phlF* operator to halt gene translation. By contrast, blue light can prevent *phlF* expression, resulting in normal translation of the regulated gene (Figure 2c). The plasmids pYF1, pEL222, and pLexRO carrying RFP fused with a YF1–FixJ–PhlF, EL222, or LexRO system were transformed into *E. coli* TOP10 for characterization. As presented in Figure 2c,d and Table 1, under blue light or darkness, YF1–FixJ–PhlF and EL222 had wide response ranges of approximately 10-fold and 7-fold, respectively. However, *sfgfp* in the LexRO system was activated in dark conditions, which indicated severe expression leakage (Figure 2e). Therefore, the optimized RBS10–CcaS#10–CcaR and YF1–FixJ–PhlF, or EL222, were used for further study. 

### 3.2. Analysis of Crosstalk of Optogenetic Parts

**Figure 3:**
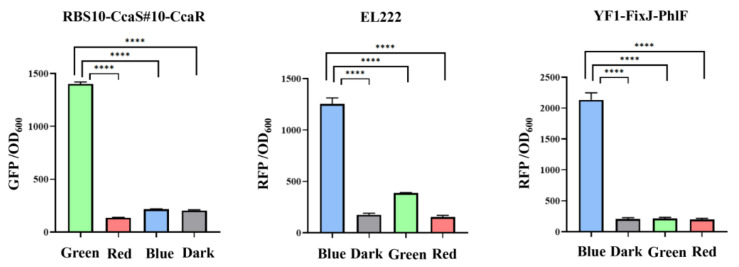
Analysis of the crosstalk of light-based systems under different lights. RBS10–CcaS#10–CcaR, EL222, and YF1–FixJ–PhlF were characterized with GFP or RFP to OD600 under single green, red, or blue lights or dark conditions. All results were calculated with three (*n* = 3) independent replications. Error bars represent means ± standard errors of the mean (SEMs). **** *p* < 0.0001.

The crosstalk of two regulation systems can prevent accurate regulation of related gene expression, which results in work turbulence in genetic circuits [35,36]. Therefore, two orthogonal optogenetic systems must be selected to create a functional optogenetic dual-switch system. Three light-based systems RBS10–CcaS#10–CcaR, YF1–FixJ–PhlF, and EL222 were expressed in the plasmids pGX, pYF1, and pEL222, respectively, and characterized by the fusion of *sfgfp* or RFP. The fluorescence intensity of *sfgfp* and RFP relative to OD_600_ was measured when the protein was exposed to a single green, red, or blue light or dark conditions. In the RBS10–CcaS#10–CcaR system, expression of *sfgfp* was up and downregulated under green and red light with a dynamic range of approximately 10-fold but demonstrated a negligible response to blue light and dark conditions (Figure 3, Table 1). These results demonstrate that an RBS10–CcaS#10–CcaR system can be incorporated into a dual-switch system. We next analyzed the EL222 and YF1–FixJ–PhlF systems in *E. coli* TOP10; the two systems were activated by blue light and repressed in dark conditions (Figure 3, Table 1). Less leakage was observed in the EL222 system under green light (quantitative fluorescence intensity of 385). These results indicate that the RBS10–CcaS#10–CcaR system crosstalks with the EL222 system under green light illumination and is completely orthogonal relative to YF1–FixJ–PhlF. Therefore, RBS10–CcaS#10–CcaR and YF1–FixJ–PhlF were used in subsequent experiments.

### 3.3. Construction of Optogenetic Dual-Switch System

**Figure 4:**
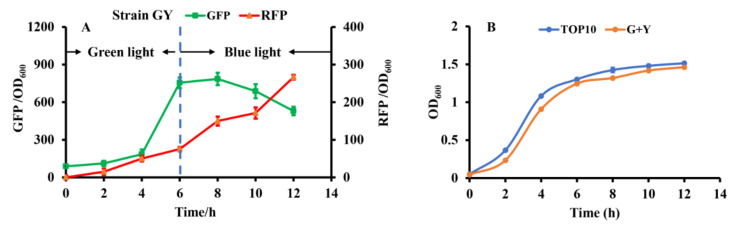
Characterizing the constructed dual-switch system under green and blue light. (**A**) Engineered strain GY containing RBS10–CcaS#10–CcaR–sfGFP and YF1–FixJ–PhlF–RFP systems. The system was irradiated with green light for hours 0 to 6 to activate GFP expression, then with blue light for hours 6 to 12 to repress GFP and activate RFP expression. (**B**) Growth of E. coli TOP10 (without light-based system) and GY. All results were calculated with three (*n* = 3) independent replications. Error bars represent means ± standard errors of the mean (SEMs).

To construct the independent dual-switch system without crosstalk, we introduced the optimized RBS10–CcaS#10–CcaR and YF1–FixJ–PhlF systems. The engineered strain GY containing the plasmids pGX (consisting of RBS10–CcaS#10–CcaR–sfGFP) and pYF1 (consisting of YF1–FixJ–PhlF–RFP) was used for characterization. The fluorescence intensity of the reporter *sfgfp* and RFP was monitored to characterize the systems. As presented in Figure 4a, the dual-switch system was initially exposed to green light for 6 h, and the fluorescence intensity of *sfgfp* was higher than that of RFP. The results indicate that the RBS10–CcaS#10–CcaR–sfGFP system was activated under green light. The green light was then switched to blue light for the 6th–12th hours of cultivation. RFP fluorescence increased at this stage, indicating that the other light-regulated system, YF1–FixJ–PhlF, is activated under blue light. Green fluorescence decreased during hours 6–12, possibly because the RBS10–CcaS#10–CcaR–sfGFP system was inhibited under blue light; however, the cell biomass increased. Additionally, the engineered strain GY demonstrated similar growth to that of *E. coli* TOP10 (without plasmid) (Figure 4b). These findings indicate that the combination of optogenetic RBS10–CcaS#10–CcaR–sfGFP and YF1–FixJ–PhlF–RFP enables highly independent regulation. 

### 3.4. Dynamically Regulating Engineered E. coli for Enhancing PHB Production with Optogenetic Dual-Switch System

**Figure 5:**
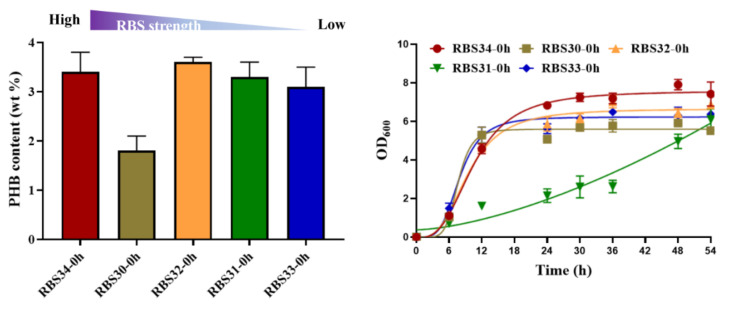
Regulation of gltA expression affected PHB production and cell growth. RBS strength of B0034 (1), B0030 (0.6), B0032 (0.3), B0031 (0.07), and B0033 (0.01) were applied for gltA expression in strains RBS34, RBS30, RBS32, RBS31, and RBS33, respectively, in accordance with the guidelines of Registry of Standard Biological Parts (http://parts.igem.org/Main_Page/ accessed on 10 March 2021). Constitutive expression of gltA and phbCAB was regulated by switching on green and blue light at 0 h. Growth curves reflect growth during 54 h of fermentation. wt% represents mass percentage of intracellular PHB to dry cell weight. All results were calculated with three (*n* = 3) independent replications. Error bars represent means ± standard errors of the mean (SEMs).

To investigate its potential for metabolic regulation, we tested the ability of the optogenetic dual-switch system to balance cell growth and improve PHB production. Citrate synthase (encoded by *gltA*) initializes the TCA cycle by catalyzing the conversion of acetyl-CoA and oxaloacetate into citrate, which affects the central metabolism of a cell [37]. Therefore, to regulate cell growth using a light-triggered system, we replaced the native promoter of *gltA* with P*_cysG2_* in the *E. coli* TOP10 genome. The expression of *gltA* was controlled using the green light-activated RBS10–CcaS#10–CcaR system (expressed in plasmid pDe–GX). To produce PHB, the *rfp* in the plasmid pYF1 was replaced with the pathway gene operon *phbCAB* to form the plasmid pYF1–PHB (Figure 1). We first examined the influence of *gltA* expression on PHB production. Different levels of RBS intensity, namely, B0030, B0031, B0032, B0033, and B0034, were used to express *gltA* in the strains RBS30, RBS31, RBS32, RBS33, and RBS34, respectively, which were transformed with the plasmids pDe–GX and pYF1–PHB. As presented in Figure 5, although the strains RBS34 (3.4 wt%), RBS32 (3.6 wt%), and RBS31 (3.3 wt%) achieved similar PHB production, RBS34 was more effective (relative to RBS32 and RBS31) with the constitutive expression of *gltA* and *phbCAB*, which was induced by switching on the green and blue light during inoculation. This finding indicated that the expression levels of *gltA* influenced PHB production and cell growth. 

**Figure 6:**
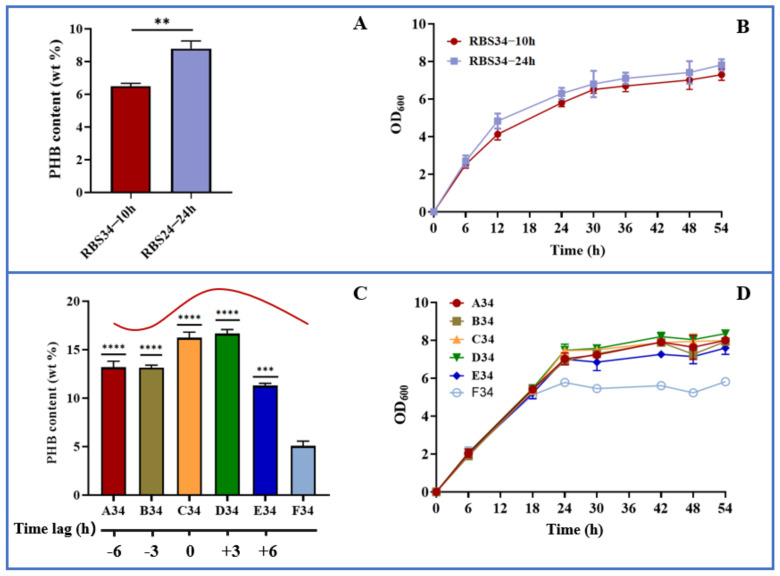
Dynamic regulation of cell growth and PHB production using optogenetic dual-switch system. (**A**,**B**) gltA repression and phbCAB activation were simultaneously regulated in strain RBS34 during two growth phases (at 10 and 24 h) by using red and blue light. (**C**,**D**) In strain RBS34, phbCAB was activated at 24 h, and gltA was repressed at 18 (A34, time lag of −6 h), 21 (B34, time lag of −3 h), 24 (C34, time lag of 0 h), 27 (D34, time lag of +3 h), or 30 h (E34, time lag of +6 h). All results were calculated with three (*n* = 3) independent replications. Error bars represent means ± standard errors of the mean (SEMs). ** *p* < 0.01, *** *p* < 0.001, **** *p* < 0.0001.

To test the dynamic regulation of *gltA* and *phbCAB*, we next analyzed the effect of expression time on PHB production by using the engineered strain RBS34. Here, a two-stage fermentation strategy (defined as the separation of cell growth and PHB production) was adopted because our previous studies verified this to be an effective strategy for improving PHB production [26,27,28]. In the present study, the *gltA* and PHB pathways were simultaneously regulated during the early (10 h) and later (24 h) log phases. Figure 6a indicates that repressing *gltA* and activating *phbCAB* expression at 24 h produced PHB content of up to 8.8 wt%, which was approximately 35% higher than that achieved at 10 h. Furthermore, the engineered strain regulated at 24 h had achieved better growth than that regulated at 10 h (Figure 6b). These results indicate that separating cell growth and PHB production during the later log phase is more beneficial for PHB production relative to separation during the early log phase. To further improve PHB production, the time lag between *gltA* and *phbCAB* was regulated. The PHB pathway was activated at 24 h. The repression of *gltA* was conducted in several growth phases, that is, 18 h (A34, time lag of −6 h), 21 h (B34, time lag of −3 h), 24 h (C34, time lag of 0 h), 27 h (D34, time lag of +3 h), and 30 h (E34, time lag of +6 h). The results are presented in Figure 6c,d, which indicate that relative to the expression of *gltA* and *phbCAB* (F34) at 0 h, the dynamic regulation of *gltA* and *phbCAB* through the variation in time lag substantially increased PHB production. The delaying of *gltA* repression until 27 h (D34) resulted in the most PHB being produced, that is, approximately 16.6 wt% or 3-fold that produced by F34 (5.1 wt%). Because PHB is an intracellular chemical, greater biomass results in enhanced PHB accumulation [38]. D34, which allows for better cell growth relative to other time lag variations, is beneficial for PHB production. Furthermore, these results revealed that the premature or late repression of *gltA* led to less PHB being produced relative to D34, which might upset the balance of acetyl-CoA flowing into the TCA and PHB pathways. In summary, our results indicate that the optogenetic dual-switch system can be easily fine-tuned to regulate cell growth and PHB production. In addition to the PHB fermentation, this optogenetic dual-switch system may be applicable to other production pathways requiring two-stage fermentation. For example, this system has potential to be applied in regulating glycerol oxidation and reduction pathways to coordinate cell growth and 3-hydroxypropionic acid production [39]. Regulating cellular carbon distribution and gene expression is conducive to product synthesis. This optogenetic dual-switch system can be utilized to regulate aspartate ammonia lyase (AspA) and aspartate aminotransferase (AspC) expression [40] or rebalance carbon substrates between pyruvate and oxaloacetate [41] for efficiently producing L-homoserine and L-threonine.

## 4. Conclusions

In the present study, a system with a greater sensing range (RBS10–CcaS#10–CcaR) relative to the CcaS#10–CcaR system was constructed by optimizing the RBS intensity of CcaS. We designed an orthogonal optogenetic dual-switch system using RBS10–CcaS#10–CcaR and YF1–FixJ–PhlF and applied this system to improve PHB production by regulating cell growth and PHB production. High expression of *gltA* in the strain RBS34 led to rapid growth and higher PHB content. To dynamically regulate the PHB pathway, separating cell growth and PHB fermentation in the stationary phase (RBS34 at 24 h) increased PHB production by up to 8.8 wt% and resulted in greater biomass. The dynamic regulation of cell growth and PHB production was analyzed for the first time by using various sequences and intervals of *gltA* and *phbCAB* expression. The results revealed that a time lag of 0–3 h was optimal for PHB production, resulting in 16.6 wt% more PHB and the greatest cell growth. The optogenetic dual-switch system in this study is a convenient tool for dynamically regulating PHB production.

## Figures and Tables

**Table 1:** Characteristics of various photosensory systems.

Property	Photosensory System			
	RBS10–CcaS#10–CcaR	EL222	YF1–FixJ–PhlF	LexRO
Sensory light	Activation in green light Repression in red light	Activation in blue light Repression in darkness	Activation in blue light Repression in darkness	Activation in blue light Repression in darkness
Photosensory range	10-fold	7-fold	10-fold	1.3-fold
Leakage	Negligible	Fewer leakage in green light	No leakage	—

## Data Availability

All data used or analyzed during this study are included in this article and the Supplementary Materials.

## Supplementary Materials

The following supporting information can be downloaded online. Figure S1: Equipment of photosensory proteins’ characterization in 24-well microassay plate (A) and shake-flask fermentation (B). Table S1: Plasmids used in this study. Table S2: Strains used in this study. Table S3: Primers used in this study. References [30,32] are cited in the supplementary materials.

## Sample Availability

Samples of the bacteria are available from the authors.
