# Supplementary material for: Development of Optogenetic Dual-Switch System for Rewiring Metabolic Flux for Polyhydroxybutyrate Production
Source: Molecules. 2022 Jan 18;27(3):617. doi: 10.3390/molecules27030617 (PMC8838604; doi:10.3390/molecules27030617)
Supplement: Supplementary file 1 [file molecules-27-00617-s001.zip › molecules-1550641-supplementary.pdf]

Supplementary Materials

A

B

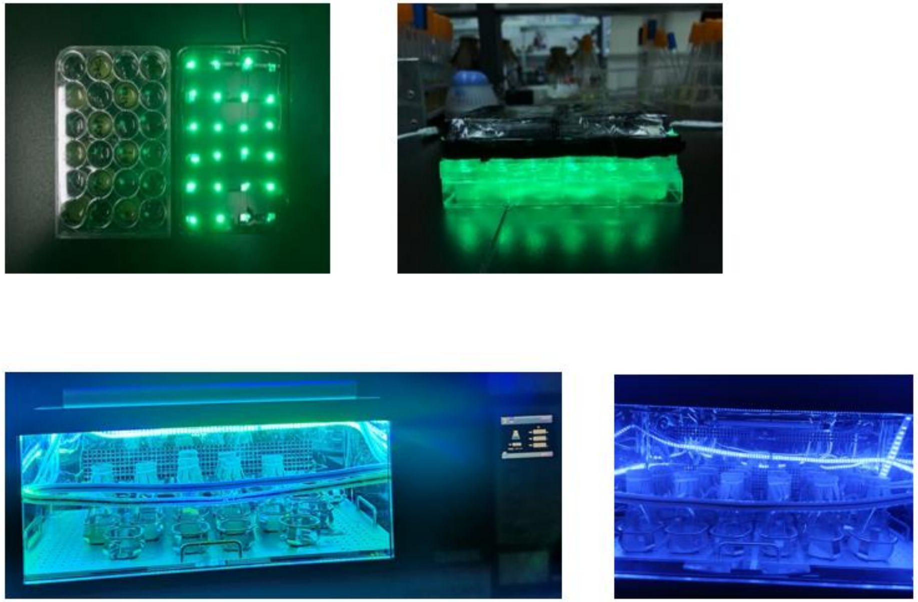

Figure S1. Equipment of photosensory proteins characterization in 24-well microassay plate (A) and shake-flask fermentation (B).

Table S1. Plasmids used in this study.

Plasmids	Description	Source
pHZ3.1(pSR58.6)	ColE1 ori, Cm^R, CcaR, *cpcG2* promoter, *sfgfp*	[30]
pSR43.6 HN (pSR43.6)	P15A ori, Sm^R, CcaS, *ho1*, *pcyA*	[30]
JFR1	ColE1 ori, Cm^R, YF1, FixJ, CcaS, CcaR, cph8, T7 core	[32]
JFR2	P15A ori, Sm^R, *phlF*, cGG, T3	[32]
pGX	P15A ori, Amp^R, ho1, pcyA, CcaS#10, CcaR, *cpcG2* promoter, *sfgfp*	This study
pYF1	ColE1 ori, Cm^R, YF1, FixJ, PhIF, RFP	This study
pLexRO	ColE1 ori, Cm^R, LexRO, RFP	This study
pEL222	ColE1 ori, Cm^R, EL222, RFP	This study
pDe-GX	P15A ori, Amp^R, *ho1*, *pcyA*, CcaS#10, CcaR	This study
pYF1-PHB	ColE1 ori, Cm^R, YF1, FixJ, PhIF, *phbCAB*	This study

Table S2. Strains used in this study.

Strains	Description	Source
E. coli DH5α	F^- supE44 ΔlacU169 (*ϕ80 lacZ ΔM15*) *hsdR*17 *recA*1 *endA*1 *gyrA96 thi*-1 *relA*1	Invitrogen
E. coli TOP10	F- mcrA Δ(mrr-hsdRMS-mcrBC) φ80lacZΔM15 ΔlacX74 nupG recA1 araD139 Δ(ara-leu)7697 galE15 galK16 rpsL(StrR) endA1 λ-	Lab Stock
#10	TOP10 carrying pGX	This study
#10-1 to #10-12	TOP10 carrying pGX with mutated RBS	This study
YF1-FixJ-PhlF	TOP10 carrying pYF1	This study
EL222	TOP10 carrying pEL222	This study
LexRO	TOP10 carrying pLexRO	This study
GY	TOP10 carrying pGX and pYF1	This study
RBS34	TOP10 *gltA*::P*cpcG2*-B0034 *gltA*, carrying pDe-GX1 and pYF1-PHB	This study
RBS30	TOP10 *gltA*::P*cpcG2*-B0030 *gltA*, carrying pDe-GX1 and pYF1-PHB	This study

RBS32	TOP10 *gltA*::P*cpcG2*-B0032 *gltA*, carrying pDe-GX1 and pYF1-PHB	This study
RBS31	TOP10 *gltA*::P*cpcG2*-B0031 *gltA*, carrying pDe-GX1 and pYF1-PHB	This study
RBS33	TOP10 *gltA*::P*cpcG2*-B0033 *gltA*, carrying pDe-GX1 and pYF1-PHB	This study

Table S3. Primers used in this study.

Primer name	Sequence (5'-3')
B3-1	CAACGGAGCTATGGACTATGTTTAAAAAACGAGAATACTATATGGCAC
B3-2	CGTCTCATTTGGACCAAAACGAAAAAAGACGCTTTTC
B3-3	CTTTTTTCGTTTTGGTCCAAATGAGACGTTGATCGGCACGTAAG
B3-4	TTTAAACATAGTCCATAGCTCCGTTGTGATGACGCATTGG
b-rfp-1	AAAGAGGAGAAATACTAGATGGCTTCCTCCGAAGACGTTATC
b-rfp-2	GGAAGCCATCTAGTATTTCTCCTCTTTACCTTAACGATACGG
b-rfp-3	GAAAACTACGCTTAACTAGAGCCAGGCATCAAATAAAACG
b-rfp-4	TGCCTGGCTCTAGTTAAGCGTAGTTTTCGTCGTTTGCTGC
GSX-1	CTCCTTTACGCATCTAGTATTTCTCCTCTTTTTAAAAATGCGATCC
GSX-4	GAGGCAGAATTTCAGAAGCTTGCATGCCTGCAGG
de-EL222-1	TGCCCCCATCTAGTATTTCTCCTCTTTCTCTAGAGC
de-EL222-2	GAAAGAGGAGAAATACTAGATGGGGGCAGACGACACA
p15A-TYB-1	GACAAGAGGAAATAGATTTCCTGGAAGATGCCAGG

RFP+EL222-1	CGGAATCTGAGCTGTTCACCATGAACAGATCGAC
EL222-F	TCTTCCAGGAAATCTATTTCCTCTTGTCAGGCCGG
EL222-R	TTTGATGCCTGGTCTAGATCAGATTCCGGCTTCGACGG
rrn-1	TATCCCCTGATTCTGTGATTTCCTCTTGTCAGGCCGG
rrn-2	CAAGAGGAAATCACAGAATCAGGGGATAACGCAGG
rfp+35v1-1	TAAAAAAGAGGAGAAATACTAGATGGCTTCCTCCGAAGACG
rfp+35v1-2	CATCTAGTATTTCTCCTCTTTTTTATTCGACTATAACAAAC
35-v1-1	AAGGGGACTGCGTTGGTGCTGGACCTCGGGGCCGACGTCGATATCTGGCGAAAATGAG
35-v1-2	GGTCCAGCACCAACGCAGTCCCCTTTGTTAGCGAAGAAAATGGTTTGTTATAGTCG
rfp-3	GAAAACTACGCTTAATAATACTAGAGCCAGGCATC
rfp-4	CTCTAGTATTATTAAGCGTAGTTTTCGTCGTTTGC
LexRO-b-1	GATGCCTGGCTCTAGTCAGCCGCGACGTTCCCA
LexRO-b-2	CGTCGCGGCTGACTAGAGCCAGGCATCAAATAAAAC
Fmcherry-1	GAGGATTTTATAATGGTGAGCAAGGGCGAGGAGGA
Fmcherry-2	CGCCCTTGCTCACCATTATAAAATCCTCTTTGACTTTTAAAACAATAAG
ori-ter-1	CATGGTGAACAGCAAGCTTGGTGTAATCATGGTCATAGC
ori-ter-2	TTACACCAAGCTTGCTGTTCACCATGAACAGATCGAC

Rmcherry-1	ATGCCTGGCTCTAGTATTACTACTTGTACAGCTCGTCCATGCC
Rmcherry-2	GTACAAGTAGTAATACTAGAGCCAGGCATCAAATAAAACG
ColE+phbCAB-F	TATGCAGGCTTCAGCTCACTCAAAGGCGGTAATACG
ColE+phbCAB-R	CTCCTCTTTCTCTAGAACCTTAACGATACGGTACGTTTCG
phbCAB-F	TCGTTAAGGTTCTAGAGAAAGAGGAGAAATACTAGCAGAG
phbCAB-R	CCTTTGAGTGAGCTGAAGCCTGCATAACGCGAAGTAATC
de-GX-4	AGTGAGCTGATACCGCAACATGAATTAACCCAGACCTG
de-GX-5	TTAATTCATGTTGCGGTATCAGCTCACTCAAAGGCGG
CTY-A1-F	CCATCATCATGTGGCGAATACCTACG
CTY-A2-R	CATCATATTCAGGAAGTTACCGGCGTAG
gltA-Kan-1	ACGATAACAACTTAGCCATGGTCCATATGAATATCCTCC
kan-FRT-R	CAATGGGCTTTGCAGCATGCAGATTGCAGCATTACAC
gltA-HA-R	TGGACCATGGCTAAGTTGTTATCGTGACCTGGATCACTG
cpcG2-F	CAATCTGCATGCTGCAAAGCCCATTGTGCTTTTCTC
gltA-F-30	ATCTAGAGATTAAAGAGGAGAAATACTAGATGGCTGATACAAAAGCAAAACTCACC
cpcG2-R-30	GTATTTCTCCTCTTTAATCTCTAGATTAAAAATGCGATCCTAACAAAGTAAAATTGAAG
gltA-F-31	AATCTAGAGTCACACAGGAAACCTACTAGATGGCTGATACAAAAGCAAAACTCACC

cpcG2-R-31	TAGGTTTCCTGTGTGACTCTAGATTAAAAATGCGATCCTAACAAAGTAAAATTGAAG
gltA-F-32	TTAATCTAGAGTCACACAGGAAAGTACTAGATGGCTGATACAAAAGCAAAACTCACC
cpcG2-R-32	GTACTTTCCTGTGTGACTCTAGATTAAAAATGCGATCCTAACAAAGTAAAATTGAAG
gltA-F-33	TTAATCTAGAGTCACACAGGACTACTAGATGGCTGATACAAAAGCAAAACTCACC
cpcG2-R-33	TAGTAGTCCTGTGTGACTCTAGATTAAAAATGCGATCCTAACAAAGTAAAATTGAAG
gltA-F-34	TTTAATCTAGAGAAAGAGGAGAAATACTAGATGGCTGATACAAAAGCAAAACTCACC
cpcG2-R-34	GTATTTCTCCTCTTTCTCTAGATTAAAAATGCGATCCTAACAAAGTAAAATTGAAG
XTYB-1	CATCATCATGTGGCGAATACCTACGA
XTYB-2	GGCGTAGGAGAGATCGTTGC

References

1 Schmidl, S.R.; Sheth, R.U.; Wu, A.; Tabor, J.J., Refactoring and optimization of light-switchable Escherichia coli two-component systems. *ACS synthetic biology* **2014,** *3*, 820–831.

2 Fernandez-Rodriguez, J.; Moser, F.; Song, M.; Voigt, C. A., Engineering RGB color vision into Escherichia coli. *Nature chemical biology* **2017,** *13*, 706–708.
